# Temporal dynamics of Middle East respiratory syndrome coronavirus in the Arabian Peninsula, 2012–2017

**DOI:** 10.1017/S0950268818002728

**Published:** 2018-10-08

**Authors:** M. A. Alkhamis, A. Fernández-Fontelo, K. VanderWaal, S. Abuhadida, P. Puig, A. Alba-Casals

**Affiliations:** 1 Department of Epidemiology and Biostatistics, Faculty of Public Health, Health Sciences Center, Kuwait University Kuwait, Khaldiya, Kuwait; 2 Department of Veterinary Population Medicine, College of Veterinary Medicine, University of Minnesota, St. Paul, USA; 3 Departament de Matemàtiques, Universitat Autònoma de Barcelona, Cerdanyola del Vallès, Barcelona, Spain; 4 Health Planning and Follow-up Department, Assistant Undersecretary Office of Quality and Development, Ministry of Health, Kuwait City, Kuwait; 5 IRTA, Centre de Recerca en Sanitat Animal (CReSA, IRTA-UAB), Campus de la Universitat Autònoma de Barcelona, 08193, Bellaterra, Barcelona, Spain

**Keywords:** ARIMA modelling, MERS-CoV, seasonality, surveillance, time-dependent reproductive number

## Abstract

Middle East respiratory syndrome coronavirus (MERS-CoV) remains a notable disease and poses a significant threat to global public health. The Arabian Peninsula is considered a major global epicentre for the disease and the virus has crossed regional and continental boundaries since 2012. In this study, we focused on exploring the temporal dynamics of MERS-CoV in human populations in the Arabian Peninsula between 2012 and 2017, using publicly available data on case counts and combining two analytical methods. Disease progression was assessed by quantifying the time-dependent reproductive number (TD-Rs), while case series temporal pattern was modelled using the AutoRegressive Integrated Moving Average (ARIMA). We accounted for geographical variability between three major affected regions in Saudi Arabia including Eastern Province, Riyadh and Makkah. In Saudi Arabia, the epidemic size was large with TD-Rs >1, indicating significant spread until 2017. In both Makkah and Riyadh regions, the epidemic progression reached its peak in April 2014 (TD-Rs > 7), during the highest incidence period of MERS-CoV cases. In Eastern Province, one unique super-spreading event (TD-R > 10) was identified in May 2013, which comprised of the most notable cases of human-to-human transmission. Best-fitting ARIMA model inferred statistically significant biannual seasonality in Riyadh region, a region characterised by heavy seasonal camel-related activities. However, no statistical evidence of seasonality was identified in Eastern Province and Makkah. Instead, both areas were marked by an endemic pattern of cases with sporadic outbreaks. Our study suggested new insights into the epidemiology of the virus, including inferences about epidemic progression and evidence for seasonality. Despite the inherent limitations of the available data, our conclusions provide further guidance to currently implement risk-based surveillance in high-risk populations and, subsequently, improve related interventions strategies against the epidemic at country and regional levels.

## Introduction

The first case of the novel Middle East respiratory syndrome coronavirus (MERS-CoV) was observed in June 2012 in Saudi Arabia [1]. Subsequently, the disease has caused more than 1500 confirmed human infections with more than 580 deaths. It spread from the Arabian Peninsula to other Middle Eastern countries from 2012 to 2018 [2, 3]. Furthermore, since 2012, MERS-CoV has jumped across continental boundaries to Europe, Northern America [4] and recently South Korea [5], which has brought global attention to the serious implications of this disease. MERS-CoV remains a notable disease and poses a significant threat to global public health, especially during mass gathering events. Thus, MERS-CoV has also become a compelling example of an emerging disease with pandemic potential due to its continuous circulation in Makkah, which hosts one of the most important global mass gathering events (i.e. the Hajj pilgrimage), where more than 2 millions of people gather from dozens of countries annually [6].

MERS-CoV causes new infections both through zoonotic and human-to-human transmission [7, 8]. Bats are implicated as the primary reservoir of the virus, but no evidence suggests a direct transmission route from bats to humans [9]. Several studies implicate Dromedary camels as the primary intermediate hosts and source of zoonotic introductions. Camel-to-human transmission appears to be through direct contact or consumption of camel products [10], while prolonged contact among human hosts is documented as the main route for human-to-human transmission [8]. Camel-to-human and human-to-human transmission have been well described in Saudi Arabia [11], while in South Korea, it is apparent that the infections are entirely human-to-human [12].

In the Arabian Peninsula, sporadic cases and small outbreaks were detected in humans in United Arab Emirates, Kuwait, Oman, Qatar, Bahrain and Yemen between 2012 and 2016 [13]. The highest numbers of cases were observed in Saudi Arabia between 2012 and 2018, which constituted more than 90% of the cases in the region [3]. The first human case of MERS-CoV was observed in June 2012 in Asir Province, located in the southwest of Saudi Arabia [14]. However, the first outbreak was not detected until 2013 in Eastern Province, with more than 70 confirmed cases, followed by Riyadh in central Saudi Arabia, with more than 60 confirmed cases [15]. The most notable outbreak was observed in the spring of 2014 with more than 500 confirmed cases in Makkah Province (western Saudi Arabia) and Riyadh [13]. To date, MERS-CoV human cases are continuously detected and confirmed every month in Saudi Arabia. Hence, the Arabian Peninsula is currently considered as the most critical global epicentre for MERS-CoV.

Some studies have suggested that observed outbreaks are seasonal [16]. However, MERS-CoV cases have been reported throughout the year, indicating a sporadic occurrence of infection. In Saudi Arabia, there was an initial increase in the number of the observed cases in the spring of 2013, which was attributed to zoonotic infection resulting from direct contact with infected young camels [7]. In the spring of 2014, the exponential increase in the number of cases was attributed to limitations in infection control and prevention within local health-care facilities [17]. Many rigorous modelling efforts were made for both periods to identify risk factors and spreading patterns of the disease on local and global levels [18, 19]. Such studies filled critical knowledge gaps in the epidemiology of MERS-CoV. However, data available from the earlier periods of the epidemic was severely lacking and suffered from many biases compared with data collected between 2015 and 2017 [20]. This is attributed to the fact that many aspects of MERS-CoV were unknown in the earlier period of the epidemic and therefore data collection was not methodical.

To date, several modelling studies have attempted to characterise both the spatial and temporal dynamics of the MERS-CoV epidemic in the Arabian Peninsula, with the ultimate goal of guiding surveillance, control and prevention activities [21, 22]. However, such studies suffered from the discrepancies and coarseness of publicly available data, which weakened the robustness of their inferences [21–23]. Inferences related to epidemic spread and introductions failed to identify evidence for seasonality. This has been attributed to either under-reporting or neglecting the significant number of asymptomatic or mild cases [21]. Unfortunately, such limitations cannot be avoided due to economic, cultural and religious issues, as well as inconsistent surveillance capabilities within the countries of the Arabian Peninsula.

Characterizing epidemic spread from surveillance data needs to account for various assumptions and uncertainties. The basic reproductive number, *R*_0_, is a commonly used parameter for quantifying epidemic spread and is defined as the average number of secondary cases produced by a primary case in a susceptible population [24]. Breban *et al*. [25] estimated the first *R*_0_ for MERS-CoV and suggested that its magnitude was comparable to a pre-epidemic phase of severe acute respiratory syndrome (SARS) in eastern Asia in 2002. Estimates about the incidence of cases and their temporal patterns are also important epidemiological parameters for measuring epidemic dynamics [26]. Recently, novel analytical methods for temporal data have been developed to model discrete time series of case incidence. Such analytical methods account for undetected cases, periodicity and under-reporting [27]. Many aspects of MERS-CoV transmission dynamics remains unknown or uncertain. For example, does *R*_0_ vary through time or across geographical regions in the Arabian Peninsula? Were there any significant seasonal components in the temporal patterns of the disease? Finally, how do the inferred estimates of MERS-CoV transmission dynamics change when including data collected from recent years?

In this study, we explore the temporal dynamics of MERS-CoV in human populations in the Arabian Peninsula between 2012 and 2017, combining two analytical methods. We quantified the spread of disease during different endemic and epidemic periods at the region and country level, and modelled the temporal dynamics of the disease during endemic periods using time series data at different spatial levels. Our objectives are to infer epidemic spread over time, identify baseline patterns of infection, and quantify seasonality and trends at different regional levels. Our ultimate goal is to present the potential of combining both methods in order to provide new insights that can help the decision-making process for allocating efficient intervention strategies and update the epidemiological knowledge about the temporal dynamics of MERS-CoV in the region.

## Material and methods

### Data source

**Fig. 1.**
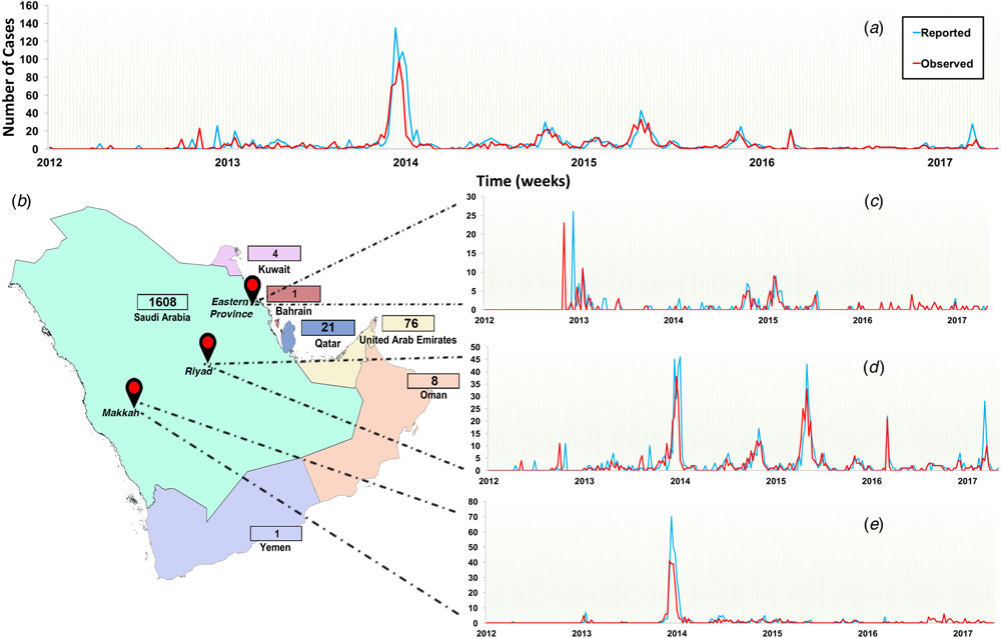
Epidemic curve of observed and reported human cases of Middle Eastern respiratory syndrome coronavirus (MERS-CoV) in the Arabian Peninsula aggregated by week from June 2012 to July 2017. (a) Epidemic of all cases observed and reported in all Arabian Peninsula countries; (b) countries of the Arabian Peninsula and their numbers of reported cases. Regional epidemic curves of observed and reported cases in (c) Eastern Province, (d) Riyadh and (e) Makkah. Numbers of reported cases are presented in the boxes adjucent to each country.

The study region included countries of the Arabian Peninsula, referred to officially as the Gulf Cooperative Council (GCC) countries, which includes Kuwait, Saudi Arabia, Bahrain, Qatar, United Arab Emirates and Yemen. We retrieved and merged data from three publicly available disease databases, namely the World Health Organization (WHO) [3], Global Animal Disease Information System (EMPRES-i) of the Food and Agriculture Organization of the United Nations (FAO) [28], and Saudi Arabia Ministry of Health web page (MoH) [2]. Thus, the final dataset compromised 1488 observed (date of case observation) and 1710 reported (date of reporting by the official government and international entities) human cases between June 2012 and July 2017 and from September 2012 to July 2017, respectively (Fig. 1a). We aggregated longitudinal data to weeks to reduce the number of time periods with zero cases within the discrete interval series and to avoid weekend effects. We then plotted and compared epidemic curves for both observed and reported cases. We used Wilcoxon signed-rank test to verify that there were no significant differences between the median of weekly observed and reported cases.

Since 95% of the cases were reported in Saudi Arabia (Fig. 1b), we further stratified our time series to plot and compare epidemic curves for three geographical regions within Saudi Arabia where the highest number of cases were observed and reported (Riyadh, Makkah and Eastern Province). Thus, our final dataset compromised four (GCC countries, Riyadh, Makkah and Eastern Province) discrete interval time series with 267 temporal units at the week level.

### Estimation of the time-dependent effective reproductive numbers

We used the likelihood-based approach utilised for the 2002 SARS epidemic, as described elsewhere [29], to estimate effective time-dependent reproductive numbers (TD-Rs) of MERS-CoV between June 2012 and July 2017 for different geographical regions. Briefly, the method averages over all potential disease transmission networks compatible with the observed cases to compute reproductive numbers for each point of time since the onset of the epidemic [29]. In this study, TD-Rs were calculated for each week of the epidemic starting from the onset week of the outbreak. We performed the analysis using the *R* package ‘*R*_0_’ [30]. As part of this process, we estimated the serial interval distribution of the generation time (GT), which is defined as the time of reporting the first primary case to the reporting of the secondary case [31]. The GT was estimated from the time lag between consecutively reported cases and its mean and standard deviation were calculated from the observed epidemic curve. We estimated the TD-Rs for the epidemic as the sum of the probabilities that a given case was the source of infection for subsequent cases based on elapsed time. We obtained 95% confidence intervals (CI) through 10 000 simulations [30]. Inferred TD-Rs with 95% CI that do not include one are interpreted as significant spreading events, while TD-Rs > 10 are interpreted as significant super-spreading events [29].

### Fitting time series using ARIMA models with trend and seasonality patterns

AutoRegressive Integrated Moving Average (ARIMA) models have become popular tools for analyzing time-series data in both veterinary and public health disciplines [32]. ARIMA models can be used for short-term forecasting of acute infectious disease incidences like influenza and hence aid in disease intervention strategies [33]. Classically, ARIMA(*p*,*q*) models are defined in the following way:1
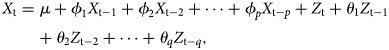
where *X*_t_ denotes the number of reported cases at time *t*, *μ* is an intercept, *ϕ*_1_,  *ϕ*_2_, …, *ϕ*_*p*_, are the coefficients of autoregressive terms, *θ*_1_, *θ*_2_, …, *θ*_*q*_ are the coefficients of moving average terms, and *Z*_t_, Z_t–1_, …, Z_t–q_ are the terms of the model which are normally distributed. These models assume that the time series is stationary and there are no trend or seasonality patterns. Usually, investigators eliminate potential linear trends and seasonality by differentiating the time series [34]. However, trend and seasonality can be included in model (1) as interpretable terms, hence avoiding losing this information. We included trend and seasonality patterns in the stationary ARIMA(*p*,*q*) model through considering the following extended model:2
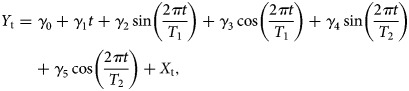
where *Y*_t_ is the observed series, *T*_1_ and *T*_2_ are different periods of seasonality of the series, the parameter *γ*_1_ captures the possible linear trend of the series, while the parameters *γ*_2_, *γ*_3_, *γ*_4_ and *γ*_5_ capture the corresponding seasonality profiles. *X*_*t*_ is the ARIMA(*p*,*d*,*q*) model expressed in (1). The trigonometric parts implemented in the model are first- and second-order Fourier terms [34], which are frequently used in time-series analysis. We estimated the parameters of the series *X*_t_, *ϕ*_1_,  *ϕ*_2_, …, *ϕ*_*p*_, *θ*_1_,  *θ*_2_, …, *θ*_*q*_ and the covariates *γ*_0_,  *γ*_1_, …, *γ*_5_ using a maximum likelihood approach. The model (1) was estimated using the arima function in R by including suitable covariates in the classical ARIMA(*p*,*q*), leading to the model (2). Three criteria were taken into account for fitting the most appropriate model: (1) the Bayesian Information Criterion (BIC), (2) the significance of the model's parameters and (3) the validation of the residuals. We selected the final model based on the balance between these three criteria.

Several abnormally large peaks corresponding to the epidemic in the selected Saudi regions were observed in the selected time-series data, between 2012 and 2017. These peaks, considered outliers in the context of classical ARIMA modelling, which consequently caused overdispersion in our selected models and severely skewed our forecasting results. Thus, we derived a threshold value to deal with from these abnormally large peaks in the time-series data. We computed this threshold through the mean plus 2.5 times the standard deviation of the sample from the series of Riyad and Makkah. As a result, thresholds of 20.86 and 20.59 cases for the Riyad and Makkah series, respectively, were obtained. The series of Eastern Province was not considered in this process due to its high frequency of zeros in its case-series data (more than 80%). Thus, we selected the threshold of 20 cases and subsequently attempted three different sensitivity analyses methods to deal such abnormally large peaks in the case-series data, as well as to assess the selected threshold of 20 cases on the forecasting results: (a) impute peaks with more than 20 cases and define them as missing values in the context of ARIMA models; (b) replacing peaks with more than 20 cases with the maximum values of the series previous to those abnormal values; and (c) replace peaks with more than 20 by the predictions provided by a linear regression in which both the trend and seasonal components were included as covariates.

## Results

### Preliminary results

The Wilcoxon signed-rank test indicated no significant difference (*P*-values <0.05) between observed and reported cases with a median difference of approximately 1.2 weeks between the date of observation and reporting (8 days). Therefore, we decided to use the reporting date series for all temporal analyses, in which the final count of cases matched those of both WHO and MoH databases for the GCC countries, because in 20.8% of cases the observation date information was missing.

Results of the sensitivity analyses for handling peaks with more than 20 cases, as described above, suggests that all of the three methods produced similar results. Therefore, we decided to select the method (a) for all of the three Saudi regions due to its simplicity and epidemiological plausibility.

### MERS-CoV epidemic spread

In Saudi Arabia, approximately 47% of reported and observed cases were in Riyadh, followed by Makkah (~26%) and Eastern Province (~12%) (Fig. 1). The epidemic curve of all observed cases in the Arabian Peninsula demonstrated a marked peak in April and May 2014, but regular small peaks afterward until 2017 (Fig. 1a). However, the epidemic curve of Eastern Province showed that the highest number of observed cases was in April and May 2013 (Fig. 1c), while the epidemic curve of Riyadh showed two large distinct peaks in spring of 2014 and fall of 2015 (Fig. 1d). The epidemic curve of Makkah showed only one distinct peak in 2014 (Fig. 1e).

**Fig. 2.**
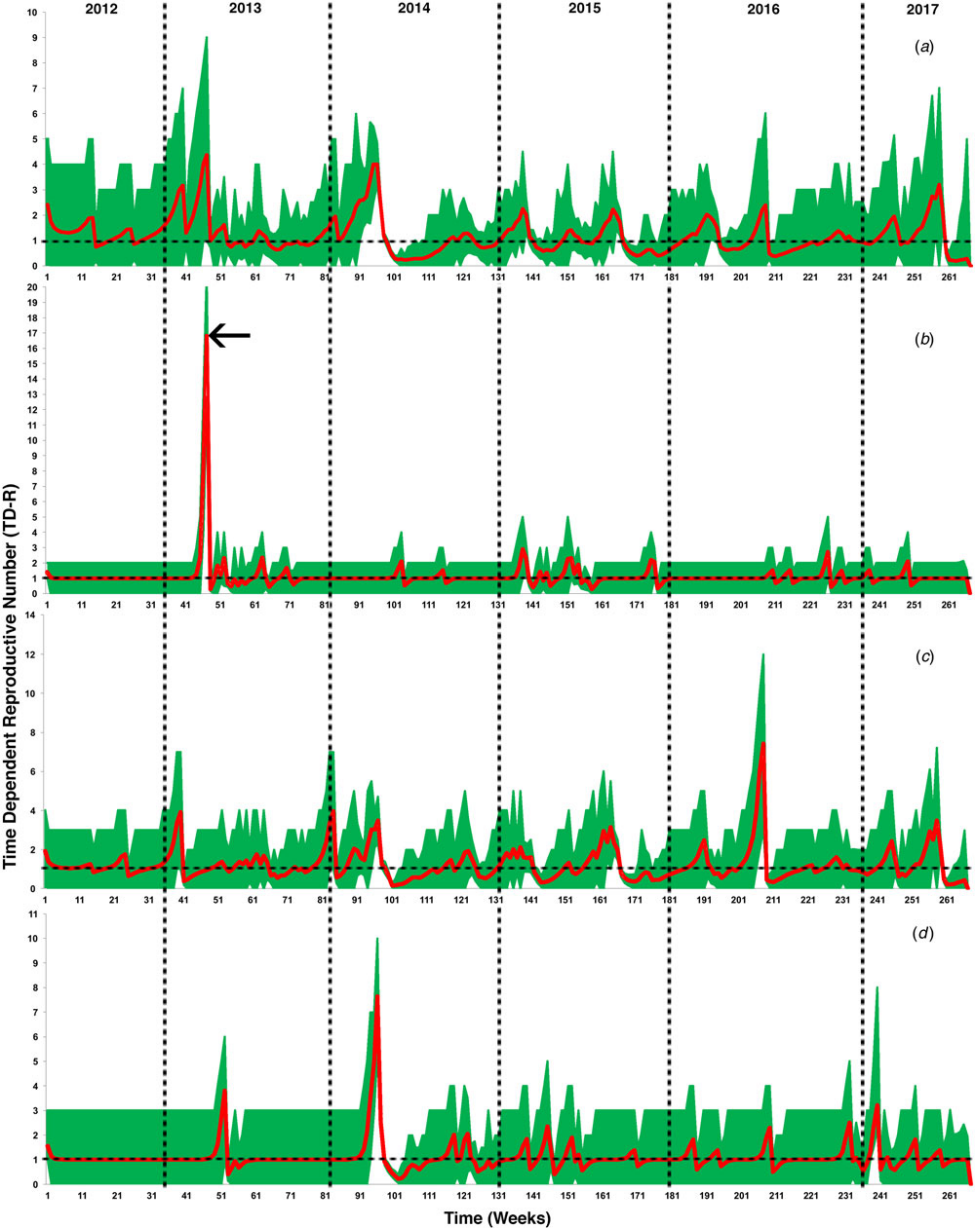
Inferred time-dependent reproductive numbers (TD-Rs) and their 95% confidence intervals (CI) for Middle Eastern respiratory syndrome coronavirus (MERS-CoV) in the Arabian Peninsula from June 2012 to July 2017. (a) TD-Rs and their 95% CI of reported cases in all Arabian Peninsula countries; (b) TD-Rs and their 95% CI of reported cases in Eastern Province; (c) TD-Rs and their 95% CI of reported cases in Riyadh; (d) TD-Rs and their 95% CI of reported cases in Makkah. The arrow in (b) indicates a super-spreading event (TD-R > 10).

Figure 2 illustrates the temporal patterns of the inferred TD-Rs throughout the study period in the selected geographical regions. On the level of the Arabian Peninsula, we inferred 12 time periods where TD-Rs were significant (TD-Rs > 1 case) (Fig. 2a and Table 1). Seven out of the 12 significant spreading events were inferred in the spring of 2014 (Fig. 2a and Table 1), while three significant spreading events inferred in 2015 (one in the winter and two in the fall; Fig. 2a and Table 1). Interestingly, our inferred estimates for the epidemic spread suggested two significant spreading events occurred in the early summer of 2017 (Fig. 2a and Table 1). We inferred three, seven and five significant spreading events in Eastern Province, Riyadh and Makkah, respectively (Fig. 2 and Table 1). In addition, one of the three inferred significant spreading events in the Eastern Province was a distinct super-spreading event (TD-R > 10) in May 2013 (Fig. 2 and Table 1). No significant TD-Rs were inferred in 2017 in Eastern Province and Riyadh (Fig. 2 and Table 1). However, two significant spreading events were inferred in January 2017 in Makkah (Fig. 2 and Table 1).

**Table 1.** Summary of significant time-dependent reproductive numbers (TD-Rs) and their 95% confidence intervals (CI) for Middle Eastern respiratory syndrome coronavirus (MERS-CoV) in the Arabian Peninsula from June 2012 to July 2017

Week	Month-year	TD-R^a^ (95% CI^b^)
*All GCC*^c^ *countries*		
92	March-2014	2.58 (1.50–3.83)
93	March-2014	2.72 (1.75–3.88)
94	April-2014	3.39 (1.67–5.67)
95	April-2014	4.00 (2.50–5.50)
96	April-2014	4.01 (3.20–4.87)
97	April-2014	2.51 (2.23–2.79)
98	April-2014	1.191 (1.01–1.33)
139	February-2015	2.03 (1.36–2.73)
165	August-2015	2.02 (1.43–2.64)
166	August-2015	1.69 (1.21–2.21)
257	May-2017	2.65 (1.37–4.10)
259	June-2017	2.23 (1.54–2.85)
*Eastern province*		
46	April-2013	8.27 (4.00–13.00)
47	May-2013	16.79 (12.00–21.00)***
139	February-2015	2.32 (1.34–3.33)
*Riyadh*		
96	April-2014	3.03 (2.11–3.89)
97	April-2014	3.47 (2.29–4.71)
163	July-2015	2.38 (1.50–3.33)
165	August-2015	2.36 (1.78–3.00)
166	August-2015	1.97 (1.47–2.47)
207	June-2016	6.02 (2.00–10.00)
208	June-2016	7.43 (3.00–12.00)
257	May-2017	2.50 (1.43–3.68)
259	June-2017	2.28 (1.70–2.83)
*Makkah*		
95	April-2014	4.98 (3.00–7.00)
96	April-2014	7.63 (5.33–10.00)
97	April-2014	2.52 (2.25–2.78)
239	January-2017	2.32 (1.50–5.00)
240	January-2017	3.21 (2.20–8.00)

a Time-dependant reproductive number.

b 95% confidence interval.

c Gulf Council Countries.

***Super-spreading event.

### Time-series analysis of weekly cases of MERS-CoV

**Fig. 3.**
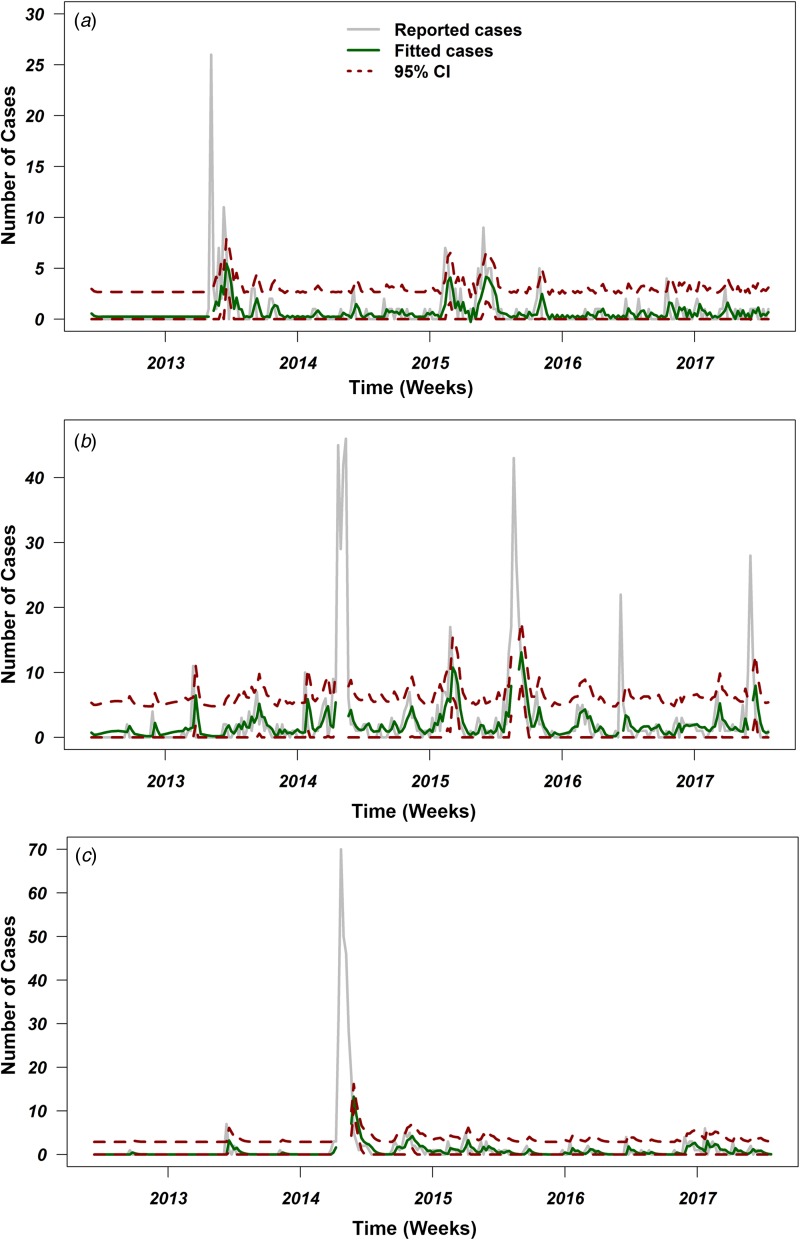
Reported cases and fitted values of ARIMA models with their 95% confidence intervals (CI) over the course of the Middle Eastern respiratory syndrome coronavirus (MERS-CoV) in Saudi Arabia from June 2012 to July 2017. Grey lines represent number of reported cases, green lines represent fitted number of cases, and red dotted lines represent 95% CI for the predicted number of cases for (a) Eastern Province, (b) Riyadh and (c) Makkah. Places where the green and red lines are discontinued represents the peaks with more than 20 cases, which have been excluded from the time-series analyses.

Figure 3 shows the reported weekly MERS-CoV cases in three selected regions of Saudi Arabia, as well as the fitted series and their corresponding 95% confidence limits. The series of Eastern Province ranges from 0 to 26 weekly MERS-CoV cases, with a median of 0 cases per week. According to the results of models (1) and (2), the best model corresponds to an ARIMA (3,0,1) leading to the equation illustrated in Table 2. The Eastern Province best-fitting model predicted a mean of one case per week and a 95% CI between zero and eight cases, suggesting an endemic pattern with no seasonality (Fig. 1a; Table 2). The Riyadh series ranges from zero to 46 cases, with a median of one case per week. The best-fitting model for the Riyadh region corresponded to an ARIMA (2,0,0) (Table 2) with biannual seasonality (*T*_2_ = 26 weeks; Fig. 3b). This selected model predicted a mean of two cases per week and a 95% CI between zero and 18 cases, suggesting a biannual seasonal pattern in the Riyadh region. Finally, the series of Makkah ranges from to zero to 70 cases per week. Similar to the Eastern Province region, the best-fitting model (Table 2) predicted a mean of one case per week and a 95% CI between zero and 17 cases (Fig. 3c), suggesting an endemic pattern with no seasonal components. The small value of standard errors for models’ coefficients and their corresponding AIC values indicate statistical robustness of the selected ARIMA models (Table 2).

**Table 2.** Summary table for the fitted ARIMA models for three major infected regions of Saudi Arabia

Region	ARIMA model (s.e. for the model's coefficients)^a^	AIC^b^
Eastern province	*X*_t_ = −0.54*X*_t−1_ + 0.56*X*_t−2_ + 0.91*X*_t−3_ + *Z*_t_ + 0.66*Z*_t−1_	881.75
	(0.08) (0.07) (0.06) (0.21)	
	*Y*_t_ = 2.00 – 0.15 sin (2*πt*/26) – 1.02 cos (2*πt*/26) + *X*_t_	1191.29
	(0.49) (0.52) (0.52)	
Riyadh	where	
	*X*_t_ = 0.50*X*_t–1_ + 0.21*X*_t–2_	
	(0.07) (0.07)	
Makkah	*X*_t_ − *X*_t−1_ = *Z*_t_ − 0.54*Z*_t−1_	947.03
	(0.08)	

a Standard errors of the model's coefficient.

b Akaike Information Criteria.

## Discussion

Over 85% of the observed MERS-CoV cases were reported in Saudi Arabia (Fig. 1b), hence our significantly inferred TD-Rs (>1 case) reflect a relatively large epidemic size, mainly on the level of Saudi Arabia, with significant progression till 2017. Our estimated regional-level TD-Rs ranged between 1.19 and 4.01 (Fig. 2a and Table 1), with the first significant TD-R inferred in March 2014 and the most recent significant TD-R inferred in June 2017. The largest TD-Rs (>3) occurred in April 2014 (Fig. 2a and Table 1), which suggested that the number of secondary cases caused by a primary case reached its maximum peak during this particular period of time [29]. That said, a super-spreading event (>10 cases) [29] was identified in May of 2013 in Eastern Province (Fig. 2b and Table 1). Furthermore, this unique super-spreading event was preceded by the largest significant non-super spreading event (i.e. approximately eight cases; Table 1) in April 2013 on the level of Saudi Arabia. Given that the highest number of cases were reported in 2014 (≈46%), this strongly suggests that epidemic progression reached its peak in 2013 and not 2014 as indicated by the non-stratified TD-R analysis and as suggested elsewhere [22]. Indeed, the 2013 super-spreading event in the Eastern province was comprised of the most notable cases of human-to-human transmission in Saudi Arabia [35]. This cluster was made up of males and females with a median age of 56 years and were all confined in a single health facility [15].

In both Makkah and Riyadh regions, the epidemic progression reached its peak (i.e. >7; Table 1) in April 2014. Those uniquely large spreading events of MERS-CoV between 2013 and 2014 in the three geographical regions of Saudi Arabia can be explained by the fact that knowledge of MERS-CoV transmission between hosts was lacking; camels were not heavily implicated as a primary intermediate host during the first 2 years of the epidemic in the region [36]. This hindered the success of control and prevention campaigns and, subsequently, led the epidemic to expand up until the first quarter of 2014. However, the role of camels in the transmission of MERS-CoV was been confirmed through the active intensive surveillance campaign conducted throughout Arabia in 2014 [37–40]. Furthermore, the development of laboratory diagnostic methods had reached a milestone in 2014, which increased the capacity for detecting of cases in both humans and animals [41, 42]. These advancements in surveillance and diagnostic methods facilitated the success of control and prevention measures in the region, which was reflected by the drop in TD-Rs after April 2014 (Fig. 2 and Table 1) [29, 43]. However, these results also suggest endemic dynamics of MERS-CoV in the region with approximately two cases per primary case between 2012 and 2017.

The Riyadh outbreaks were characterised by a large number of virus introductions with distinct genetic diversity, while Makkah outbreaks, like Eastern Province, were characterised by high intensity of human-to-human transmission [22]. These human-to-human transmissions were dominated by healthcare-associated cases [44]. Therefore, it was suggested that hospital-associated transmission had a larger role than direct contact with an infected camel in the spread and maintenance of the virus in the GCC region [44]. Thus, both regions are important epicentres for MERS-CoV spread and maintenance in the Arabian Peninsula.

Eifan *et al*. used similar incidence data reported between May 2013 and May 2015 to calculate the reproductive numbers and concluded that the impact of reported sporadic cases in Saudi Arabia was insignificant and, subsequently, suggested that there was little zoonotic influence on MERS-CoV transmission dynamics [45]. Furthermore, the study concluded insignificant progression of MERS-CoV epidemic after 2015 in multiple geographical regions within Saudi Arabia [45]. However, our inferred TD-Rs suggest a significant epidemic progression from 2013 to 2017, which sustained itself perhaps in camels to reach an endemic state in the Arabian Peninsula [38–40]. In addition, the temporal frame of region-specific TD-Rs inferred from the three major Saudi Arabian geographical areas mostly agreed with the temporal dynamics of significant TD-Rs inferred from the non-stratified analyses, discussed above (Figs 2 and 3). Furthermore, according to the WHO and Saudi MoH [2, 3], the last incident cases were detected in February and March 2018 in Saudi Arabia and Oman. One of the 2018 Saudi cases died from MERS-CoV infection due to contact with camel in the Riyadh region [2]. This confirms the notion that MERS-CoV continues to maintain itself in the camel–human transmission cycle in the Arabian Peninsula, as suggested elsewhere [46].

Our inferred TD-Rs for the three regions of Saudi Arabia did not demonstrate evidence for seasonality of MERS-CoV outbreaks (Fig. 2 and Table 1); instead, they showed erratic behaviour through the period of the present study. However, using reproductive numbers and/or observed incident case series are not valid tools for quantifying temporal patterns of infectious disease outbreaks, as demonstrated in the past MERS-CoV studies [23]. Therefore, a valid statistical presentation of seasonality is needed to properly guide intervention efforts.

In this study, our time-series analyses indicate that temporal patterns of MERS-CoV incident cases across the primary infected regions of Saudi Arabia were similar in Eastern Province and Makkah, but different in Riyadh region (Fig. 3). Our ARIMA results statistically demonstrate that biannual seasonality was evident in the Riyadh region (Fig. 3b) as shown by coefficients of the trigonometric covariates included in the model (Table 2). Indeed, Riyadh is the capital of Saudi Arabia, and like Eastern Province, is densely populated with camels and characterised by heavy seasonal camel-related activities (e.g. grazing, movement, trade and mating) [47]. Also, Riyadh hosts seasonal camel racing events from April to October, during which camels mostly come from the eastern and central regions of Arabia. During these annual racing events, camels are usually transported in large trucks from their local ranches, crossing borders to different cities and race track sites within Saudi Arabia and other GCC counties to Riyadh [48].

In Eastern Province, no statistical evidence of seasonality was identified (Fig. 3a). Instead, an endemic pattern of MERS-CoV cases, with sporadic outbreaks, was the best-fitting model for the incident case series of the Eastern Province (Table 2). Eastern Province is a major agricultural area and the most camel densely populated region on the level of the whole Arabian Peninsula. Besides, Eastern Province is the only geographical hub that is characterised by a massive network of camel movement and exchange between the countries of the GCC throughout the year [38, 47]. These characteristics led to the circulation, establishment and maintenance of an endemic state of MERS-CoV in the Eastern Province camel populations [47]. Furthermore, the annual movements of camels from Eastern province to Riyadh might constitute an important dispersal route of season zoonotic infection between human and camels [46].

Like Eastern Province, our ARIMA model for Makkah region inferred an endemic state of MERS-CoV with sporadic outbreak pattern (Table 2; Fig. 3c). This could be due to the fact that the frequency of zoonotic transmission was substantially lower in Makkah than in Riyadh and Eastern Province, and a higher percentage of the cases resulted from human-to-human transmission [22]. However, the role of annual holy pilgrimage held in Makkah (where millions of people around the world gather in a mass religious ceremony) remains controversial in the human-to-human transmission of MERS-CoV [49]. The pilgrimage season depends on the Islamic calendar, measured by moon phases, rather than the Gregorian calendar, which is more associated with annual climate seasonality. Therefore, we were not surprised that our best-fitting time-series model did not include seasonality for Makkah (Fig. 3c). Finally, there are distinct cultural and socio-economic differences between the three regions. Hence, one would expect that MERS-CoV temporal dynamics would behave differently across major regions of Saudi Arabia.

Reporting bias and lack of information on the time of implementation of intervention measures might substantially bias the estimates of our temporal analyses, and hence it is a limitation of the present study [29, 50]. Furthermore, it is expected that surveillance efforts in Saudi Arabia were mainly focused on the capital, followed by the Makkah (as the holy city of pilgrimage), where major high-end health facilities are located. Thus, outbreak data used in our study may have been biased towards regions with higher surveillance capabilities, leading to inferences skewed towards these regions, as in the case of Riyadh. This example of reporting bias has implications for the inferred temporal dynamics of the epidemic at regional levels. However, although Eastern Province had the lowest number of cases, we detected a unique super-spreading event in 2013 (Figs 2 and 3). This gives us confidence that the overall results of our temporal analyses are not skewed towards high-reporting region like Riyadh.

Another limitation of the present study is that we were unable to analyse the temporal pattern of MERS-CoV in other GCC countries. As mentioned above, the wide range of seasonal camel activities occurring within and between GCC countries should result in larger numbers of cases. This is, again, attributed to the reporting bias reflected by the inconsistent surveillance capabilities of the GCC countries, as well as related to their conservative culture and strong attachment to their camel herds [51]. Out of all the GCC countries, only Saudi Arabia provided public information through their government health page with consistent updates on the case status, while other countries only reported to international organisations, such as OIE and WHO. However, Saudi Arabia is substantially larger than other GCC countries, and therefore, epidemic magnitude and size might be proportional to the size of the affected country. It is worth noting that MERS-CoV is characterised by asymptomatic and mild cases, which pose a great challenge for healthcare practitioners in attempting to identify and diagnose cases. Hence, under-reporting bias has become another natural limitation of the present study, as thousands of cases might have been missed during the current epidemic [52]. Under-reporting bias is expected to be more severe in the earlier years of the epidemic. Finally, excluding large peaks (which represents time interval with more than 20 cases) of the epidemic curve in our ARIMA models represents another important limitation. Those peaks represent sporadic introductions, which are difficult to model due to their erratic nature. Furthermore, including such peaks causes overdispersion, which in turn may bias forecasting for future incidence of cases. That said, the number of cases forecasted by our selected ARIMA models using the time-series data between 2012 and 2017 was similar to the number of cases reported in Saudi Arabia between January and June of 2018 [2, 3], which indicate that our analytical approaches were statistically sounded.

In conclusion, this study characterises the temporal dynamics of the MERS-CoV epidemic in the Arabian Peninsula between 2012 and 2017, with the goal of assessing and guiding surveillance efforts in the region. We used two unique approaches for the analysis of epidemic curve (or time-series) data to quantify disease progression and model specific temporal patterns on a regional level. In both analytical approaches, we accounted for geographical variability between three affected regions in Saudi Arabia including Eastern Province, Riyadh and Makkah. Our inferred *TD-Rs* indicate relatively large epidemic sizes, mainly on the level of the whole country, with significant progression until 2017 (TD-Rs > 1). In both Makkah and Riyadh regions, the epidemic progression reached its peak in April 2014 (TD-Rs > 7), where most of the MERS-CoV cases were reported. However, a super-spreading event (TD-R > 10) was identified in May 2013 in Eastern province, which comprised the most notable incident cases of human-to-human transmission. Our best-fitting ARIMA model inferred statistically significant biannual seasonality in Riyadh region, a region characterised by heavy seasonal camel-related activities. However, no statistical evidence of seasonality was identified in Eastern Province and Makkah. Instead, both regions were characterised by endemic pattern of cases with sporadic outbreaks. While the data used in the present study have inherit limitations, we demonstrated that continuous modelling efforts of publicly available MERS-CoV data can play a significant role in shedding new insights into the epidemiology of the virus. These insights include new inferences about epidemic progression and evidence for seasonality. Such new epidemiological inferences may provide further guidance to currently implemented risk-based surveillance efforts in high-risk populations and, subsequently, improve related interventions strategies against the epidemic on the country and regional levels.
